# Exacerbated Skeletal Muscle Inflammation and Calcification in the Acute Phase of Infection by Mexican *Trypanosoma cruzi* DTUI Strain

**DOI:** 10.1155/2014/450389

**Published:** 2014-06-02

**Authors:** Andrea Vizcaíno-Castillo, Andrea Jiménez-Marín, Bertha Espinoza

**Affiliations:** Departamento de Inmunología, Instituto de Investigaciones Biomédicas, Universidad Nacional Autónoma de México, 04510 Mexico City, DF, Mexico

## Abstract

A murine model was used to study the histopathological aspects and cytokine expression levels in skeletal muscle provoked by the infection with Mexican TcI strains. BALB/c mice were inoculated with the virulent Querétaro strain and the nonvirulent Ninoa strain. Parasite numbers were counted in blood and skeletal muscle at different times post-infection, and real time-PCR expression levels of the cytokines IL-12, IL-4, IL-10, IFN-**γ**, and TNF-**α** were evaluated. In the acute phase of infection, a high parasitic load, both in blood and skeletal muscle, was detected. The histopathological analyses showed an exacerbated inflammation and granulomatous-like infiltrate with the Querétaro strain. Interestingly, extensive calcification areas were observed in the skeletal muscle surrounded by inflammatory infiltrates. TNF-**α** and IL-10 expression exhibited a significant increase at the peak of infection. In summary, Querétaro strain, a Mexican TcI strain, is virulent enough to induce high inflammation and calcification in skeletal muscle of the hind limbs, which could be related to high expression levels of TNF-**α**.

## 1. Introduction

Chagas disease, an important health problem in Latin America, is the manifestation of tissue damage resulting from infection with the hemoflagellate protozoan parasite,* Trypanosoma cruzi*. This parasite constitutes a very heterogenic taxon, allowing the clustering of* T. cruzi* strains into six discrete typing units (DTUs): TcI to TcVI [1]. The DTU most abundant and dispersed in the Americas is TcI, which can be associated with domestic and sylvatic cycles and, in human infection, with cardiomyopathy [2]. Several studies have shown that* T. cruzi *genetic heterogeneity is related to the progression and severity of Chagas disease. In South America, strains belonging to* T. cruzi *II group (currently known as TcII to VI DTUs) have been mainly associated with the domestic/peridomestic cycle and hence with the development of this illness [3]. Although DTUI (TcI) dominate the sylvatic cycle of the Amazonia area, some countries from the northern region of South America, such as Venezuela and Colombia, have demonstrated the predominance of TcI strains in human [4–6]. In Mexico, it has been reported that 98% of the isolated strains belong to TcI and are closely related to each other [7, 8]; the presence of chronic symptomatic evolutions has also been shown [9–11], with 5000 people affected with severe chronic chagasic cardiomyopathy [12], indicating that TcI is dominant in Mexico even in human infections.

*T. cruzi *can infect many different cell types, but most strains show a preference for growth in muscle tissue forms. Here, the acute infection elicits a variety of immune effectors mechanisms characterized by the participation of major immune cells such as macrophages and T-cells, as well as the production of several proinflammatory (IL-12, TNF-*α*, and IFN-*γ*) and regulatory (IL-4, IL-10) cytokines [13–15]. However, these responses not only permit the control of the parasite, but also triggered a strong inflammatory reaction that can result in severe tissue damage. Studies in experimental models of Chagas disease have shown myofibrosis and myositis as well as degeneration and necrosis of myofibres [16, 17], although the extent and severity of the lesions can vary depending on the animal strain [18] or the* T. cruzi* strain [19].

Severe inflammation has been considered as an important feature of tissue calcification in some clinical conditions. Two mechanisms of calcification are recognized: metastatic calcification (elevated levels of calcium and/or phosphate in serum that produce systemic mineralization) and dystrophic calcification (associated with injury, infection, or rheumatic diseases with normal calcium/phosphate homeostasis) [20, 21]. Dystrophic calcification is also related to cellular death, leading to catabolic enzymes and calcium release. In cases of aortic calcification, tissue mineralization has been observed near to inflammatory infiltrates, and degradation products from apoptosis can promote it [22]. Some studies have shown association of the cytokine tumor necrosis factor alpha (TNF-*α*) with vascular calcification [23, 24]. This cytokine is present in both acute and chronic inflammation and, in chronic cases, can induce the activation of apoptotic pathways [22].

The different biological and genetic properties of this intracellular parasite contribute to a wide diversity in infectivity, virulence, pathology, and tissue parasitism in a broad range of mammalian hosts [25]. There are a vast number of research papers related to the pathogenesis and histology of* T. cruzi* infections, but most of them used* T. cruzi* II parasites or are conducted in South American countries. Thus, the genetic and biological characterization of circulating TcI strains in Mexico is necessary. In our search for characterization of Mexican strains, we have found biological differences among them, in spite of their genetic closeness [8, 9], and more recently we described the immunological response of mice to virulent and nonvirulent Mexican strains [26, 27].

In the present work, we continue this search by studying the skeletal muscle from mice infected with two Mexican TcI strains, named Ninoa and Querétaro, with regard to parasitism and inflammatory response, and cytokines expression. Our data showed differences in parasitemia and tissular parasitism between these strains. Remarkably, infection with the Querétaro strain triggered an exacerbated inflammatory reaction, which led to skeletal muscle injury and important calcification of the tissue particularly during the acute phase. Besides, the infection with this strain induces higher expression levels of the cytokines TNF-*α* and IL-10, compared to Ninoa infection, that could be related to the tissue damage observed.* In vitro* studies also demonstrated a higher release of TNF-*α* by macrophages infected with the Querétaro strain. To our knowledge, this is the first report showing calcification phenomenon in mice skeletal muscle of the hind extremities due to* Trypanosoma cruzi* TcI infection.

## 2. Materials and Methods

### 2.1. Parasites

The Mexican* T. cruzi* strains, Querétaro and Ninoa, both belonging to DTU TcI [7, 8] were used for this study. Ninoa (MHOM/MX/1994/Ninoa) strain was isolated from an acute human Chagas disease patient in Oaxaca, Mexico [28]. Querétaro (TBAR/MX/0000/Querétaro) strain was isolated from an insect vector,* Triatoma barberi*, at Querétaro, Mexico. The strains were maintained by serial passage in BALB/c mice; bloodstream trypomastigotes were obtained by cardiac puncture of these animals (collected in heparinized vials) and used for inoculations of the experimental mice.

### 2.2. Mice and Experimental Groups

Seven- to eight-week-old female BALB/c mice weighing 18–20 g were obtained from our animal facilities (Animal House of the Instituto de Investigaciones Biomédicas, from the Universidad Nacional Autónoma de México, IIB-UNAM). This study was approved by the ethics committee for animal experimentation of the IIB-UNAM. All procedures and experimental protocols were performed according to Biosafety committee of the IIB-UNAM for the use of animals in experimental conditions. Two groups of 12 animals each were intraperitoneally infected with 10^4^ blood trypomastigotes in 200 *μ*L of sterile PBS (Querétaro group and Ninoa group); the control group (12 animals) was inoculated with the same volume of PBS. Parasitemia levels were determined by counting the number of parasites present in a 5 *μ*L blood sample collected from the tail vein every third day. Three animals from each experimental group were sacrificed at 1, 15, 21 (acute phase), and 90 days (chronic phase) after inoculation (pi) and the skeletal muscle from the hind leg were obtained, as previous studies have shown it as a target tissue of this parasite. Two independent experiments were carried over (a total of 24 animals per group).

### 2.3. Histopathological Analysis

Half of the skeletal muscle obtained from each animal, either infected or control mice, were fixed in 4% paraformaldehyde and paraffin-embedded for histopathological analysis. Tissue sections (5 *μ*m thick) were deparaffinized and stained with hematoxylin-eosin (H-E). Five nonconsecutive slides (sections separated by 50 *μ*m) per animal were examined under a light microscope (Optiphot-2, Nikon), and pictures were taken using a Coolpix 4300 camera (Nikon). The proportion of tissue area presenting inflammatory cells was estimated over the total sections of the tissues analyzed, using the inflammation scores described by Sun and Tarleton for skeletal muscle: 0 = normal, 1 = scarce cellular infiltrate, 2 = diffuse infiltrate, 3 = abundant infiltrate, and 4 = granulomatous-like infiltrate [29].

Additionally, sections were treated with the von Kossa stain in order to verify the presence of calcium deposits. Briefly, deparaffinized slides were treated with 1% aqueous silver nitrate solution under UV light for 30 minutes, washed with distilled water, and then treated with a solution of 5% sodium thiosulphate for 5 minutes (to remove the unreacted silver). The slides were counterstained with nuclear fast red for 5 minutes.

### 2.4. Immunohistochemistry

Sections from the paraffin-embedded tissues were deparaffinized and rehydrated through several ethanol baths (absolute, 96%, 70%, and 50%) and water in order to perform immunohistochemical studies to evaluate tissue parasitism and detect macrophages in the inflammatory infiltrates. Briefly, the hydrated tissues were blocked with 2% bovine serum albumin in phosphate-buffered saline (BSA/PBS) for 2 h at room temperature in a moist chamber in order to block unspecific unions. Slides were then incubated overnight at 4°C with a biotinylated F4/80 antibody, a macrophage-specific antibody (1 : 50; Caltag Laboratories, CA, USA) and a polyclonal rabbit anti-*T. cruzi *serum (1 : 1000; serum was previously obtained in our laboratory using a total extract from the Querétaro strain). Slides were then washed and incubated for 60 min with a mix of streptavidin-phycoerythrin (SA-PE, 1 : 500; Caltag Laboratories) for the detection of macrophages and a fluorescein-labelled anti-rabbit IgG (anti-IgG-FITC, 1 : 100; Sigma Immuno Chemicals, MO, USA) for the detection of parasites. After washing 3 times with PBS, the slides were counterstained with DAPI 1 : 1000 (Molecular Probes, Oregon, USA) and mounted. Preimmune serum and IgG2b antibody (Caltag Laboratories), the same isotype of the F4/80 antibody, were used as negative controls. The stained slides were visualized using a Zeiss fluorescence microscope. By this technique, the number of parasite nests (tissue parasitism) was counted using the 40x objective in 60 fields per mouse in skeletal muscle. In the same slides, the number of fields with presence of macrophages was counted as well as the number of infiltrated nests.

### 2.5. Total RNA Extraction and cDNA Synthesis

The other half of the tissues obtained was used to extract total RNA. Isolated skeletal muscles were frozen immediately in dry ice and stored at −70°C until use. RNA extraction was performed using TRIzol reagent (Invitrogen, Carlsbad, CA, USA), following the recommendations of the manufacturer. Integrity of RNA was verified by agarose gel electrophoresis.

Target RNA (2 *μ*g) was treated with 2 U of recombinant DNase I (Kit DNA-*free*, Ambion, Inc., USA) in a final volume of 20 *μ*L, as recommended. Five hundred ng of DNase treated-RNA were reverse transcribed using 1.25 U/*μ*L MultiScribe RT, 0.4 U/*μ*L RNase Inhibitor, 2.5 *μ*M oligo d(T)_16_, 0.5 mM dNTPs, 2.75 mM MgCl_2_, and 1x Taqman RT buffer, in a total volume of 20 *μ*L (TaqMan Reverse Transcription Reagents kit; Applied Biosystems, CA, USA). The reaction proceeded for 10 min at 25°C, followed by 30 min at 48°C, and finally 5 min at 95°C in a PTC-100 Programmable Thermal Controller (MJ Research Inc.). The single-strand cDNA synthesized was stored at −20°C until its use.

### 2.6. Real-Time PCR Amplification

PCR reactions were performed in the ABI Prism 7000 SDS (Applied Biosystems) using the SYBR Green PCR kit. Each amplification reaction was performed in a final volume of 20 *μ*L, containing 1x SYBR Green PCR Master Mix, 300 nM of each primer (reverse and forward), and 50 ng of single-strand cDNA sample. PCR conditions were as follows: 1 cycle at 95°C for 10 min and 40 cycles at 95°C for 15 sec, 60°C for 1 min. A no-RT control (DNase-RNA treated without being reverse transcribed) for each sample and a negative control (without DNA sample) for each primer pairs were included in each PCR reaction set.

The target genes analyzed included IL-4 (FWD: GCAGAGACTCTTTCGGGCTTT; REV: TCATTCATGGTGCAGCTTATCG), IL-10 (FWD: GGAAGACAATAACTGCACCCACTT; REV: CCGCAGCTCTAGGAGCATGT), IL-12 (FWD: CGTGCTCATGGCTGGTGCAAAG; REV: CACATGTCACTGCCCGAGAGT), IFN-*γ* (FWD: AATGAACGCTACACACTGCAT; REV: TGGCAGTAACAGCCAGAAACA) and TNF-*α* (FWD: GGGCAGGTCTACTTTAGAGTCATTG; REV: GGCTGGGTAGAGAATGGATGAA), and the constitutive gene HPRT (hypoxanthine phosphoribosyl transferase; FWD: GAAAGACTTGCTCGAGATGTCA; REV: AGCACACAGAGGGCCACAA) was used to normalized the data. The primers were designed using the Primer Express Applications-based primer design software (Applied Biosystems, CA, USA). Also, the capability of HPRT gene as an internal reference gene for our model was verified (its expression is not affected by* T. cruzi* infection).

In order to obtain the amplification efficiencies for each gene, standard curves were elaborated by using 21 days pi Querétaro-infected-mouse skeletal muscle cDNA serial dilutions (range 1–100 ng). Each dilution was amplified with every primer pair. Ct values were plotted against the logarithm of initial cDNA concentration. Amplification efficiency of each gene was calculated from the linear equations obtained, with the formula: *E* = 10^−1/slope^ [30].

Real-time PCR results were analyzed using the Q-Gene software, of free access at http://www.biotechniques.com/ [31]. This program takes into account the amplification efficiencies of the genes to calculate the normalized gene expression of the target gene (with respect to a reference gene) and compares its relative expression to the control group.

### 2.7. *In Vitro* Assays

Trypomastigote forms of* T. cruzi* Ninoa and Querétaro strains were obtained from infected mice and maintained in Vero cells cultures grown in D-MEM medium (GIBCO) supplemented with 10% fetal bovine serum (FBS) and incubated at 37°C, 95% relative humidity, and 5% CO_2_. Culture supernatants were collected for parasites harvesting and centrifuged at 800 g for 10 min; the pellet was left undisturbed for 30 min, in order to allow the alive trypomastigotes to swim towards the supernatant and eliminate the most of cellular debris. Then, the supernatant was collected and centrifuged at 2000 g for 10 min.

Macrophages J774 (2 × 10^6^ cells per 25 cm^2^cellular culture bottle) were cultured previously in D-MEM supplemented with 10% FBS with or without 100 U/mL IFN-*γ* at 37°C, 95% relative humidity, and 5% CO_2_. After, cells were infected with trypomastigotes at a parasite-to-cell ratio of 7 : 1 for Querétaro strain or 15 : 1 for Ninoa strain, according with previous infection assays performed in our laboratory (data not shown) and incubated for 24 h. Production of cytokines IL-10, IL-12, and TNF-*α* was measured from supernatant cultures using IL-10 Mouse ELISA kit (ENDOGEN), IL-12 Mouse ELISA kit (ENDOGEN), and TNF-*α* Mouse ELISA kit (ENDOGEN), following manufacturer's instructions. Duplicate measures from three independent experiments were carried out.

### 2.8. Statistical Analysis

The data are expressed as the arithmetic mean ± standard deviation of six mice for each experimental point from two independent experiments. The differences between the groups were determined by Student's *t*-test (for parasitemia, histological analyses, and* in vitro* assays) or by one-way ANOVA with Bonferroni posttest, considering *P* < 0.05 as statistically significant (for real-time PCR analyses).

## 3. Results

### 3.1. Clinical Disease

**Figure 1:**
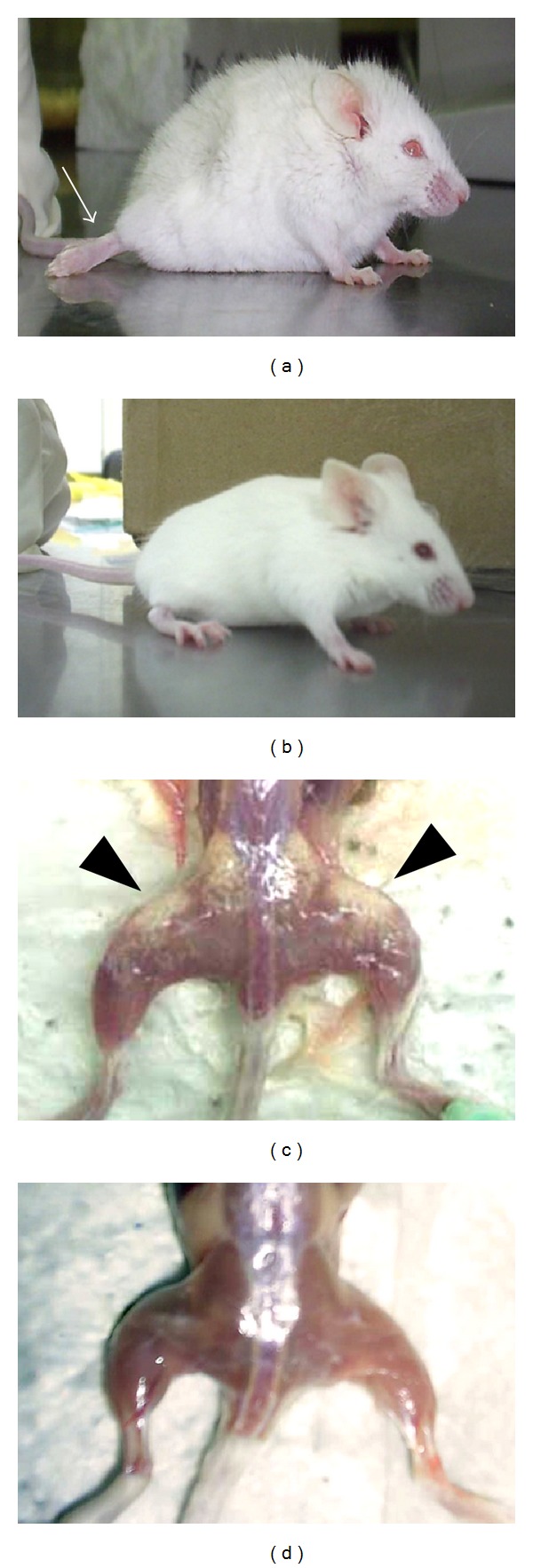
Macroscopic characteristics of a mouse infected with 10 000 trypomastigotes of Querétaro strain ((a), (c)) and a control mouse ((b), (d)). The Querétaro strain infection provokes back bristling hair and loss of mobility of their posterior extremities ((a); arrow). After sacrifice and removal of the skin, the skeletal muscle of mouse infected with Querétaro strain showed pale areas on the tissue ((c); arrowheads).

**Figure 2:**
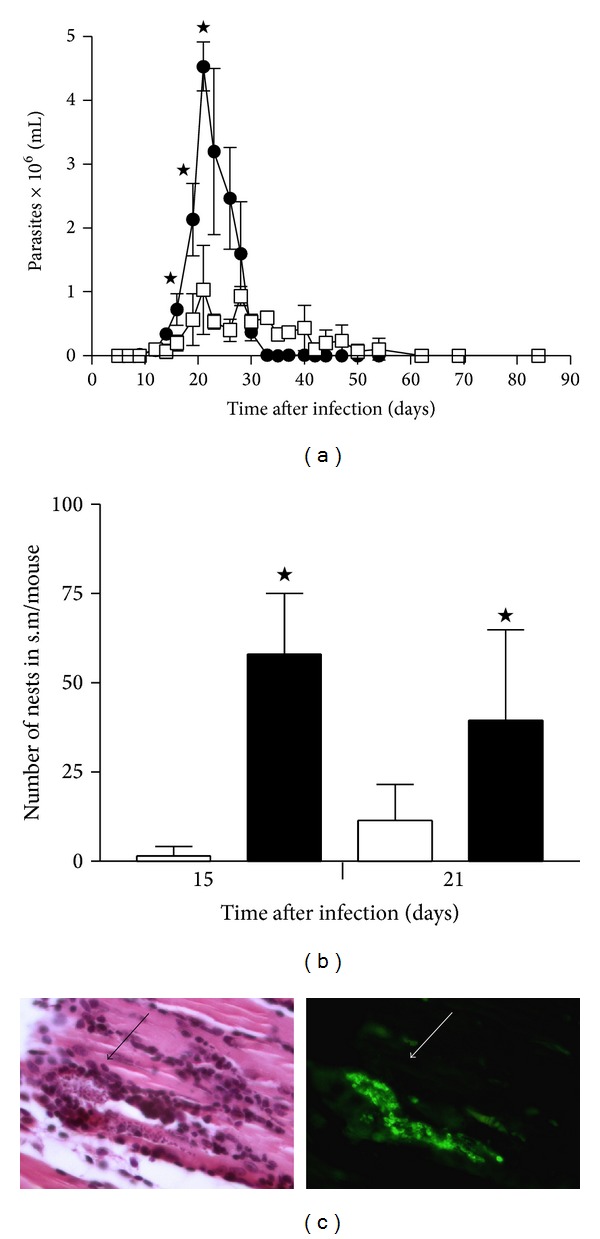
Kinetic of infection of two Mexican* Trypanosoma cruzi* strains in blood and skeletal muscle. (a) Number of blood parasites during the infection with Querétaro (●) or Ninoa (□) strains. Data represent average ± SD of at least three mice by experimental point. A representative graph of two independent experiments is shown. ^★^ *P* < 0.05 with Student's *t*-test. (b) Number of nests of parasites in skeletal muscle (Querétaro, solid; Ninoa, cleared). Data represent average ± SD of 6 mice from two independent experiments. ^★^ *P* < 0.05 with Student's *t*-test. (c) Microphotograph of the same field stained with H-E (left) and immunofluorescence (right) of a nest in s.m. infected with Querétaro strain, 21 dpi. Magnification 400x. (s.m. = skeletal muscle).

The animals infected with the Querétaro strain showed back bristling hair, a continuous tremor in their whole body, and loss of mobility of their rear extremities by days 13–15 pi (Figure 1(a)). These manifestations were coincident with the increasing in the parasitic load (Figure 2(a)), but they recovered some mobility of the hind legs by 90 days pi. On the contrary, neither the mice infected with Ninoa strain (not shown) nor the uninfected control mice (Figure 1(b)) showed these characteristics.

An important difference between both infections was the presence of pale areas (chalky white patches) observed only on the skeletal muscle of the posterior extremities from the animals infected with the Querétaro strain (Figure 1(c)), as well as loss of muscular mass in the same area. These pale areas were still seen until 90 days pi.

### 3.2. Parasitemia

The circulating blood parasites were counted every third day from 5 to 90 days pi. Parasitemia become detectable by day 12 pi and reached the peak by day 21 pi with 4.5 × 10^6^ parasites/mL for the Querétaro infection, being significantly higher than with the Ninoa infection by almost 5-fold increase (*P* < 0.05). Then, the number of parasites decreased until reach undetectable levels by day 35 pi with the Querétaro strain, but during the Ninoa infection the parasites became undetectable until day 60 pi (Figure 2(a)).

### 3.3. Tissue Parasitism

The parasitic load in skeletal muscle was examined over the course of infection in mice with each strain of* T. cruzi*. The amastigote nests were detected by immunohistochemistry and counted (Figure 2(c)). By day 15 pi the number of nests in Querétaro group was 19.6 ± 7.6, whereas in Ninoa group it was only 0.5 ± 1.2, meaning a 35-fold difference between strains (*P* < 0.05; Figure 2(b)). By day 21 pi, a higher number of amastigote nests was still seen during Querétaro infection (12.9 ± 10.8) in relation to Ninoa infection (3.9 ± 3.4), with a significant 3.3-fold difference between strains (*P* < 0.05). In the chronic phase of infection (90 day pi), tissue parasites were neither detected in Querétaro nor in Ninoa strain infection.

### 3.4. Inflammatory Infiltrates

**Figure 3:**
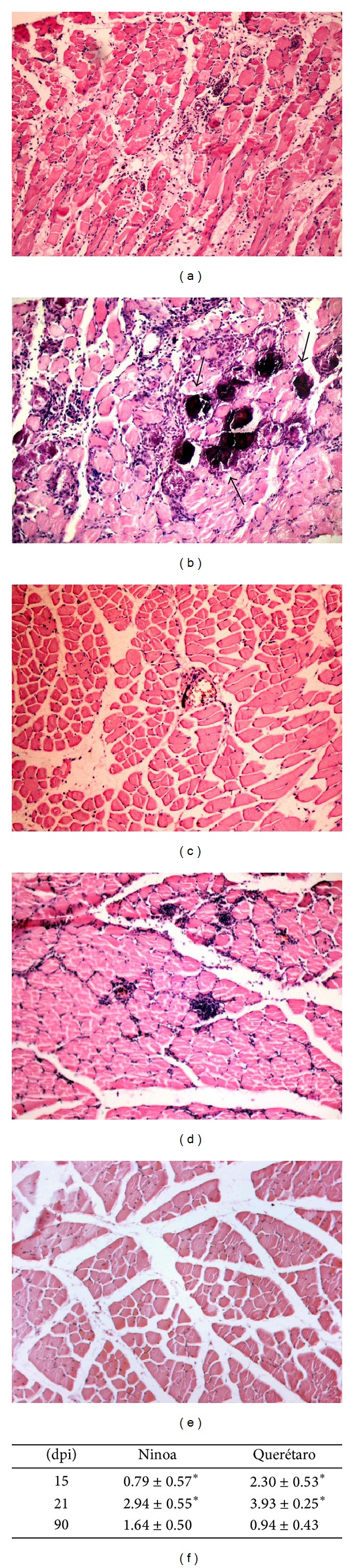
Histopathological analysis of* Trypanosoma cruzi* infection in skeletal muscle. (a) Querétaro, 15 days pi; (b) Querétaro 21 days pi; note the intense basophilic stain areas (arrows); (c) Ninoa, 15 days pi; (d) Ninoa, 21 days pi; (e) Control. Magnification 100x. (f) Inflammation scores (arbitrary units) for Querétaro and Ninoa infections. Data represent average ± SD of 5 nonconsecutive slides per animal of 6 mice in two independent experiments. *P < 0.001, with Student's *t*-test.

Infection with both* T. cruzi* strains induced inflammatory infiltrates in the skeletal muscle which was analyzed in slides stained with H-E; such inflammation could be observed as of 15 days pi (Figures 3(a) and 3(c)). At the peak of infection, the tissue sections obtained from the Querétaro group had intense interstitial inflammatory infiltrate with profuse cells on muscular fibers with granulomatous-like infiltrates, as well as disorganization and myofibers necrosis (Figure 3(b)). In contrast, sections of muscle in Ninoa infected mice presented clusters of inflammatory cells, observed at interstitial spaces, with mild tissue damage (Figure 3(d)). At the chronic phase of infection (90 dpi), the inflammatory infiltrates were scarce in both groups (data not shown).

Infiltrates in Querétaro group at day 15 pi were 2.30 ± 0.53, and 3.93 ± 0.25 for the 21 day pi, whereas for Ninoa infection they were 0.79 ± 0.57 at 15 days pi and 2.94 ± 0.55 at 21 days pi (Figure 3(f); arbitrary units), according to the score described in Material and Methods. These scores represented a statistically significant difference of 3- and 1.3-fold for 15 and 21 days pi, respectively (*P* < 0.001). At 90 days pi, the inflammation diminished and the score was similar for both strains.

**Figure 4:**
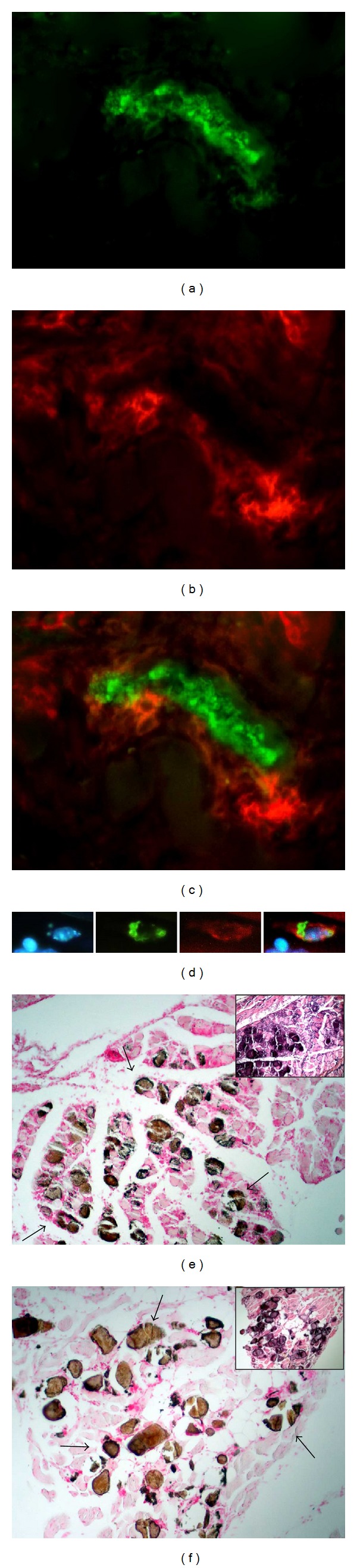
Macrophages and calcification areas in skeletal muscle due to Querétaro strain infection. (a) Immunofluorescence of a large amastigote nest in skeletal muscle of mice infected with Querétaro strain, 21 days pi. (b) Same field showing immunofluorescence of macrophages surrounding the nest. (c) Merge of (a) and (b). Magnification 400x. (d) Microphotographs of amastigotes (green) inside a macrophage (red); counterstained with DAPI. Extreme right: merge. Zoom of a magnification 400x. (e) Calcification areas stained in brown (arrows), 21 days pi. (f) Calcification areas (arrows) at 90 days pi. The inserts show calcification areas stained with H-E. Magnification 100x.

The histological analyses showed that most of the inflammatory infiltrates were composed mainly of mononuclear cells. To identify the presence of macrophages, double immunofluorescence techniques to simultaneously stain macrophages and parasites were used. A large number of cells in these infiltrates were macrophages and some of them were associated with amastigote nests (Figures 4(a)–4(c)). Apparently, intracellular amastigotes were also found in macrophages (Figure 4(d)). Moreover, many macrophages appeared closed to calcification areas. At chronic phases, some macrophages were still seen (data not shown).

### 3.5. Tissue Calcification

An interesting finding was the presence of sites with intense basophilic stain exclusively in skeletal muscle from animals infected with Querétaro strain (arrows, Figure 3(b)). In order to determine the presence of calcium deposits in these basophilic areas, sections were treated with the von Kossa stain, making evident that stained areas corresponded to calcification sites, as shown in Figures 4(e)-4(f): the dark brown-stained areas indicate tissue calcification (arrows). More calcification areas were observed during the peak of infection, and these areas can still be seen at 90 days pi, along with regeneration of the muscle, which is seen as holes between fibers indicating the possible substitution of myofibers by adipose tissue (Figure 4(f)). Remarkably, none of these calcification areas were seen on skeletal muscle from Ninoa-infected mice or control mice at any time after infection, as neither H-E nor von Kossa staining revealed any calcinosis area (data not shown).

### 3.6. Cytokines Profile in Skeletal Muscle

**Figure 5:**
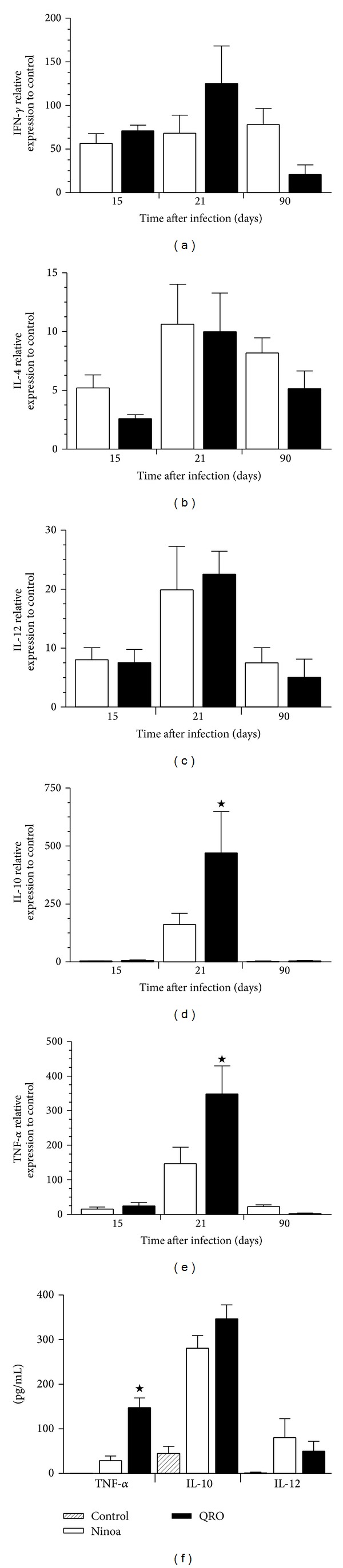
Cytokines determination in skeletal muscle from mice infected with two Mexican* Trypanosoma cruzi *strains (Ninoa, cleared bars; Querétaro, solid bars; Control, stripped bars). mRNA expression levels were quantified by qPCR: (a) IFN-*γ*, (b) IL-4, (c) IL-12, (d) IL-10, and (e) TNF-*α*. Data are expressed as average ± SEM of 6 mice per experimental group from 2 independent experiments. ^★^ *P* < 0.05 with ANOVA test and Bonferroni posttest. (f) Cytokine concentration (pg/mL) of* in vitro* infections of macrophages J774 with each TcI strain. Data are presented as average ± SD of double measures from three independent experiments. ^★^ *P* < 0.05 with Student's *t*-test.

To assess the expression levels of some cytokines following* T. cruzi* infection, real-time PCRs were used to quantify the mRNA expression profiles of IL-4, IL-10, IL-12, IFN-*γ*, and TNF-*α* within the skeletal muscle at days 1, 15, 21, and 90 pi. In general, the overall expression of each cytokine in both infections was similar with detectable transcripts present at day 15 pi, peaking at day 21 and decreasing by day 90 (Figure 5). Only the IL-10 and TNF-*α* transcripts showed a significant difference (*P* < 0.05) between both strains at the peak of infection (Figures 5(d) and 5(e), resp.), being higher with the Querétaro strain but decreasing dramatically by 90 days pi.

In* in vitro* assays, a higher production of TNF-*α* was induced in macrophages infected with the Querétaro strain, even without previous activation with IFN-*γ* (Figure 5(f)).

## 4. Discussion

In previous studies, our research group has shown the important presence of* T. cruzi *TcI strains in Mexico [7, 8]. However, the published information on biological and histopathological characteristics of the Mexican TcI strains is scarce.

In this study, we analyzed the histopathological damage of skeletal muscle from mice caused by the infection with the Mexican TcI strains, Querétaro and Ninoa. Animals infected with Querétaro strain presented back bristling hair, a continuous tremor in their whole body, and stiffness of the hind limbs, manifestations that are not shown by Ninoa-infected mice. In a previous study, looking for a molecular diagnosis method to study tropism and growth kinetics of* T. cruzi* in a murine model, similar characteristics in mice infected with another Mexican isolation (JALGO, obtained from Triatominae feces collected at Jalisco, Mexico) were reported, and the animals succumbed to the infection; however, the genetic group of this strain was not reported [32].

As previously described [26, 27], the magnitude of the parasitemia at the pick during the Querétaro infection was higher than with the Ninoa infection. The number of parasites detected in blood correlated with the number of amastigote nests detected in the skeletal muscle, being significantly higher in animals infected with the Querétaro strain. The detection of tissular parasites was made by immunohistochemistry, facilitating the localization of amastigote nests, mainly of small ones or those covered by inflammatory cells. These results together indicate that Querétaro strain has a higher ability to invade and replicate into the host organism than Ninoa strain.

*T. cruzi* infection provoked an inflammatory phenomenon mainly composed of mononuclear cells. An increase in the intensity and extension of the inflammatory infiltrates was observed, and the peak of inflammation correlated with the peak of parasitic load, at 21 days pi. Querétaro strain induced severe focal and diffuse inflammatory foci with necrosis of the tissue and granulomatous-like infiltrates, whereas, in comparison, Ninoa strain provoked a moderate inflammation. These results support the previous observation that the characteristics of each strain promote differences in evasion of the host immune response that could be responsible for higher parasitic loads and, in consequence, more intense* in situ* inflammatory reactions, as has been suggested by Garzon and collaborators [33]. In contrast, at the chronic phase of infection, where no parasites were detected, inflammatory foci have diminished, indicating that the host immune response has controlled the infection.

The analysis also revealed an interesting finding of* T. cruzi* pathogenesis: the presence of calcium deposits in the areas with intense basophilic stain, exclusively observed during the Querétaro infection. These areas corresponded with the pale zones macroscopically observed (shown in Figure 1) from where the samples were taken. The chalky white patches of the zone reminded an illness of the cattle known as white muscle disease, which is consequence of tissue calcification, and is also associated with stiffness of the hind limbs [34]. The deposited calcium contributes to the white color of the affected musculature. In this study, the presence of calcium deposits was verified by dark brown-stained areas after treating slides with the von Kossa staining. Tissue calcification can be the result of tissular damage involving death cell or tissular proteins denaturalization that allow the precipitation of calcium salts (dystrophic calcification). This sort of calcification can also be associated with unspecific lesions of degenerative or necrotic type. For example, in animal models of calcification induced by cardiotoxin injection, it has been suggested that calcification is preceded by death cell of the injured tissue, with disruption of the skeletal architecture [21]. In our model of infection, calcification seems to be coincidental with the exacerbated inflammation, indicating a relationship between the efforts to control the infection and the development of the lesion. Thus, the extensive and necrotic damage observed could be the reason of the calcinosis produced with the Querétaro strain infection. Considering the damage that calcification produce in muscular tissue, the paralysis of posterior extremities that Querétaro infected mice showed could be a consequence of the calcinosis process, perhaps due to the destruction of muscular architecture. There are some reports that mention this paralysis [32, 35], but it has been only recently that Ramirez-Archila and coworkers [36] confirmed that contractile properties of skeletal muscle are impaired during infection with TcI parasites, and these properties remained attenuated as a consequence of the replacement of the muscular fibers by fibrous tissue and fat. The same authors found calcifications in the skeletal muscle studied, but they studied the rectus abdominis muscle. A recent study reports the presence of skeletal muscle fibers undergoing calcification and necrosis as a consequence of* T. cruzi* II infection in WT and Daf1-deficient mice [37]. There is another report that superficially mentions muscular calcification as a consequence of* T. cruzi* infection, but that study was achieved using also a genotype II strain [38]. To our knowledge, the present work is the first report of tissue calcification in skeletal muscle from the posterior extremities induced by a* T. cruzi *TcI infection.

An important effector cell involved in this parasite control is the macrophage. Immunofluorescence techniques showed abundant presence of macrophages surrounding amastigote nests as well as areas with calcification and tissular damage. The presence of macrophages has been demonstrated in inflammation areas closest to mineralized tissue in calcific aortic valves in humans [39]. Besides, it has been reported that macrophages can enhance osteogenic signals elicited by vascular smooth muscle cells, thus playing a significant role in the formation of plaque calcification [40]. Although the incidence of macrophages in the inflammatory infiltrates was higher, other lymphocytic cellular types were present. A previous report indicated the presence of macrophages, plasmatic cells, and some eosinophils at the infiltrates in skeletal muscle of mouse infected with Ninoa strain [28]. In the present study, it was not possible to determine the exact nature of other cellular types because of the technique selected. It is known that CD4- and CD8-antigens are labile and is recommended to use freeze-tissue sections for their detection [29]. More studies are needed, in order to obtain a more complete image of the cells controlling the parasite replication in skeletal muscle during a* T. cruzi* I infection.

We then became interested in the cytokines expressed in infected skeletal muscle, since the cytokine profile during an infection can be crucial for its outcome. A balance between proinflammatory and regulatory cytokines is necessary in order to control the infection [41]. Hence, the expression of proinflammatory and regulatory cytokines was evaluated by real-time PCR. An important finding was the elevated levels of TNF-*α* mRNA during the acute phase of infection with Querétaro strain. It has been shown that macrophages in inflammatory infiltrates of calcific aortic valves express TNF-*α* [42] and that calcification process may be regulated by mechanisms involving the presence of this cytokine [43]. The significant increment in TNF-*α* levels could be associated with the histological findings of this work, particularly those of calcification. Besides, elevated concentrations of TNF-*α* were also detected when macrophages* in vitro* were infected with the virulent strain. Interestingly, the infection alone was capable of activating the macrophages J774 and inducing the production and release of this cytokine.

Therefore, the high expression of TNF-*α* could be part of the mechanisms involved in the calcinosis process and the severe tissular damage observed in the skeletal muscle during Querétaro infection. It has been reported that an exacerbated inflammatory response in this sort of infection, involving proinflammatory cytokines such as IL-12, IFN-*γ* and, particularly, TNF-*α*, contribute not only to control the infection but also to damage the host tissue. For example, TNF-*α* presence has been shown in necrotic areas in spleen from* T. cruzi* infected mice [44]. Accordingly, the intense inflammatory reaction observed in Querétaro infected animals could have caused a necrotic damage of the muscle ending in its calcification.

In the present study it was also notable the upregulation of IL-10, a cytokine that possess a downregulatory activity on the development and effector functions of cell-mediated immunity. Previous studies have shown that infection of IL-10^−/−^ mice with the protozoan parasite* T. cruzi *resulted in a reduced parasitemia but caused increased mortality [45, 46], possibly indicating a more systemic immune response which, if not regulated by IL-10, results in a toxic overproduction of proinflammatory cytokines, like TNF-*α* [47]. In this way, the increased levels of IL-10 found in* T. cruzi*-infected skeletal muscle may be a necessary event for controlling the strong cell-mediated immunity elicited by this parasite.

In conclusion, the most relevant finding of the present work was the development of calcification in the skeletal muscle of hind legs, exclusively with Querétaro strain and particularly during the acute phase of infection, even though both strains studied belong to DTU I. This calcification event may probably be a consequence of the exacerbated inflammatory process and the consequent disruption of the tissue elicited by this infection and could be related to a significant increment in TNF-*α* levels.
